# Improving Clinical and Family Communication for Adult Child Caregivers of a Parent With a Blood Cancer: Single-Arm Pre-Post Pilot Intervention

**DOI:** 10.2196/38722

**Published:** 2022-07-05

**Authors:** Carma L Bylund, Easton N Wollney, Gemme Campbell-Salome, Allison J Applebaum, Samantha R Paige, Kennan DeGruccio, Elisa Weiss, Maria Sae-Hau, Jason Arnold, Domenic Durante, Tithi B Amin, Chelsea N Hampton, Carla L Fisher

**Affiliations:** 1 Department of Health Outcomes & Biomedical Informatics University of Florida Gainesville, FL United States; 2 Geisinger Danville, PA United States; 3 Memorial Sloan Kettering Cancer Center New York, NY United States; 4 STEM Translational Communication Center University of Florida Gainesville, FL United States; 5 School of Social Work Columbia University New York, NY United States; 6 The Leukemia & Lymphoma Society Rye Brook, NY United States; 7 E-Learning, Technology and Communications University of Florida Gainesville, FL United States; 8 Department of Advertising University of Florida Gainesville, FL United States

**Keywords:** caregiver, clinician-patient communication, healthy communication practice, eHealth literacy, family communication, feasibility, acceptability, oncology, blood cancer, cancer patent, web-based information seeking, health information, clinical communication, smartphone, mobile phone

## Abstract

**Background:**

Adult child caregivers of parents with cancer may face challenges when communicating with the patient and other family members, communicating during clinical interactions, and navigating web-based information seeking.

**Objective:**

We developed and pilot-tested the *Healthy Communication Practice* program for adult child caregivers of parents with a blood cancer, which aims to help participants learn and implement communication skills central to caregiving. We assessed the feasibility and acceptability of the training.

**Methods:**

Eligible participants completed a preprogram survey. We assessed the feasibility of participants completing the intervention in the allotted time. Participants had 2 weeks to complete the 2-part, 90-minute online program and completed a postprogram survey that included program evaluation items and the Acceptability of Intervention Measure (AIM) using a 1-5 rating scale (5=strongly agree).

**Results:**

Of 50 caregivers who initially expressed interest, 34 consented, and 30 completed the program and both surveys (88% completion rate). Caregivers had a mean age of 45.07 (SD 11.96) years and provided care for parents who had a mean age of 73.31 (SD 9.38) years. Caregivers were primarily daughters (n=22, 73%). Overall, scores on the AIM scale were high (mean 4.48, SD 0.67). Specifically, caregivers felt the content met their communication needs (mean 4.58, SD 0.62) and their own needs as a caregiver of a parent with a blood cancer (mean 4.39, SD 0.72).

**Conclusions:**

We demonstrated the feasibility and acceptability of the *Healthy Communication Practice* program, which aims to enhance family and clinical communication skills among caregivers of a parent with a blood cancer. Future studies will examine the efficacy of the program and its impact on both caregiver and patient communication and health outcomes.

## Introduction

Family caregivers of individuals diagnosed with cancer face many challenges as they integrate the emotional, logistical, and financial pressures of cancer caregiving into their often busy lives [1,2]. Over the past 2 decades, researchers have developed psychosocial interventions to address some of the challenges that cancer caregivers face [3]. However, all caregivers are not the same. One key differentiating characteristic of caregivers is their relationship to the individual with cancer. Family caregiver-patient dyads generally represent 3 caregiver types: an individual caring for their spouse, child, or parent. The third type of caregiver, adults caring for an aging patient, receives the least attention in research [4], despite the expectation that they will increase in number due to population shifts and the forthcoming “silver tsunami” [5]. In addition, research suggests that adult child caregivers of parents, particularly daughters, can experience higher levels of strain [6], stress [7], guilt [8], and burden [9] compared to those caring for a spouse.

Furthermore, there has been little research on caregiving for patients with a hematologic or blood cancer. There are unique challenges associated with blood cancer caregiving among both acute and chronic blood cancer disease subtypes, and families facing a blood cancer diagnosis can be at a higher risk for psychological distress compared to those coping with other types of cancers [10,11]. The median age at diagnosis for the most common types of blood cancer (leukemia and non-Hodgkin lymphoma) is 67 [12,13]. Given that aging adults are more likely to be coping with a blood cancer, it is not uncommon for midlife adult children to become the primary caregiver of a parent diagnosed with a blood cancer [1]. Midlife caregivers report significant stress and burden related to family functioning, which can be heightened when juggling care for multiple generations. Many midlife caregivers must manage multiple roles in addition to caring for their parent, including demands within their own families, homes, and professional lives [1].

Communication is a central component of caregiving. In our previous work [14-16], we identified communication challenges that adult children face when caring for a parent with a blood cancer that are uniquely complex, given their role and the relational shift that occurs when they take care of a parent who used to care for them [16]. Furthermore, our previous work demonstrates that these adult child caregivers commonly report caregiving communication skills deficits in navigating cancer information in web-based and clinical settings and in facilitating open and supportive communication within the family [14,16,17].

Therefore, we developed a web-based intervention, the *Healthy Communication Practice* program, to help adult children caring for a parent with a blood cancer develop and implement communication strategies that can improve their caregiving experience (Multimedia Appendix 1). The program was designed to take approximately 90 minutes to complete and be accessible across multiple platforms including computers, tablets, and smartphones. The specific aims of this study are as follows:

- To examine the feasibility of the Healthy Communication Practice program among adult children caring for a parent with a blood cancer.
- To examine the acceptability of the Healthy Communication Practice program among adult children caring for a parent with a blood cancer.

## Methods

### Study Design

We conducted a single-arm, pre-post pilot study of a web-based communication intervention at the University of Florida.

### Ethics Approval

The University of Florida Institutional Review Board approved the study (202101030). All participants provided consent before the preintervention survey.

### Intervention

The *Healthy Communication Practice* is a self-paced, web-based program developed for adult children who care for a parent, parent-in-law, or stepparent currently living with a blood cancer (eg, leukemia, lymphoma, myeloma). Grounded in communication and education theories [18-25] and based on our extensive preliminary work (in-depth interviews and surveys) with cancer caregivers [14-17], this program teaches caregivers essential communication skills in eHealth literacy, clinical encounters, and family relationships. We teach concepts and skills to help caregivers navigate web-based cancer information, communicate with their parent’s doctors, find meaning in their caregiver role, and use open and supportive communication to strengthen relationships and facilitate communication within their family. To achieve these aims, the program is divided into 2 parts: (1) navigating cancer information in web-based and clinical settings; and (2) facilitating open and supportive communication in the family. Participants could stop the program when needed and return later without losing their progress.

We developed the program in collaboration with experts in web-based education and instructional design (JA and DD). We used a variety of instructional techniques including experts introducing concepts and skills; authentic caregiver narratives; video demonstrations of clinical and family encounters; and interactive activities designed to keep participants engaged such as writing, reflection prompts, and quizzes. An advisory board was formed consisting of 3 oncologists and 2 caregivers, and a clinical oncology social worker provided feedback on the program before the pilot test. The process of working with the advisory board allowed us to ensure we had presented realistic caregiving scenarios and correct medical advice.

### Recruitment and Procedures

We recruited participants through The Leukemia & Lymphoma Society [26], the International Waldenstrom’s Macroglobulinemia Foundation, and ResearchMatch. To be eligible to participate, individuals had to reside in the United States, be 18 years of age or older, and be providing care for a parent, parent-in-law, or stepparent with a blood cancer at the time of recruitment. Their parent had to be currently living, diagnosed at least 3 months prior to inclusion (in order for them to have experience caregiving), and either in treatment or had treatment completed within the last year. Participants who met the criteria for the study and provided consent were given questionnaires at 3 different points in time: (1) a preprogram survey before the start of the program, (2) a postprogram survey within 1-7 days of program completion, and (3) a postprogram survey 3 months after the completion of the program. This paper reports only on data from the pre- and postprogram surveys. All screening and questionnaire data were collected online using REDCap. All potential participants received study information via email. As such, we inferred that they had access to a device and internet to complete the study. Data collection on the pre- and postsurveys took place between June 2021 and January 2022.

Participants who screened into the study were immediately directed to the preprogram survey, which contained demographic and other questions (Table 1) including the consent form. The first author then sent participants an individualized link to the *Healthy Communication Practice* program. The unique link allowed us to know when a participant started the intervention, monitor their progress, and know when they finished the intervention. In addition, the unique link allowed the participants to log back in whenever they wanted, with their progress having been saved. Participants were given 2 weeks to complete the program, and up to 2 reminders were sent as needed. Upon completing the program, we sent participants an immediate postprogram survey followed by a US $75 e-gift card.

**Table 1:** Demographics of caregivers and their parents.

Characteristics			Values (N=30)
Caregiver age (years), mean (SD)			45.07 (11.96)
Caregiver age (years), min-max			24-67
Parent age (years), mean (SD)			73.31 (9.38)
Parent age (years), min-max			57-89
**Relationship type (caregiver to parent), n (%)**			
	Daughter, daughter-in-law, or stepdaughter	22 (73)	
	Son, son-in-law, or stepson	8 (27)	
**Relationship type (parent to caregiver), n (%)**			
	Mother, mother-in-law, or stepmother	16 (53)	
	Father, father-in-law, stepfather	11 (37)	
	Unreported	3 (10)	
**Children, n (%)**			
	Caregivers with children	12 (40)	
	Caregivers with children under 18 years of age	6 (20)	
**Siblings, n (%)**			
	Caregivers with siblings	22 (73)	
	Caregivers with 1 sibling	7 (23)	
	Caregivers with 2 siblings	8 (27)	
	Caregivers with 3 siblings	6 (20)	
	Caregivers with 4 siblings	1 (3)	
**Race^a^, n (%)**			
	White	24 (80)	
	Black or African American	4 (13)	
	Asian	5 (17)	
	Native Hawaiian or Pacific Islander	1 (3)	
	American Indian	0 (0)	
**Ethnicity, n (%)**			
	Hispanic	5 (17)	
	Non-Hispanic	25 (83)	
**Education, n (%)**			
	High school graduate or General Education Diploma	3 (10)	
	Some college degree	1 (3)	
	2-year degree	2 (7)	
	4-year degree	11 (37)	
	Master’s degree	10 (33)	
	Doctoral degree	2 (7)	
	Professional degree	1 (3)	
**Employment status, n (%)**			
	Employed full time	17 (57)	
	Employed part time	4 (13)	
	Self-employed	3 (10)	
	Not employed	3 (10)	
	Retired	3 (10)	
**Relationship status, n (%)**			
	Single or never married	9 (30)	

^a^Caregivers were allowed to select more than 1 option.

### Measures

Prior to conducting the study and based on previous research [27,28], we determined that the intervention would be deemed feasible if 70% of consented participants completed the intervention within the allotted 2-week time period and the postsurvey within the allotted 1-week time period. We recognize that there are various ways of defining feasibility [29-31], but for the purposes of this study, we chose to use completion as was done in a previous caregiver intervention [27]. Our decision was based on *Healthy Communication Practice* being a newly developed intervention and our primary concern being whether people would complete it in the given time, rather than how we would recruit them. We assessed acceptability of the program using the Acceptability of Intervention Measure (AIM) [32]. This is a 4-item measure scored on a 5-point Likert scale (1=strongly disagree, 5=strongly agree). Items asked whether the *Healthy Communication Practice* program met participants’ approval and met participants’ needs, whether the participants liked the program, and whether they welcomed the program. As the AIM is still relatively new and there were no cutoff scores for it, we decided prior to the study that the intervention would be deemed acceptable if participants completing 80% or more of the intervention had mean scores of 4 or higher on the AIM items. In addition, we asked participants to rate the extent to which they felt the program was clear and met their needs as an adult child caregiver of a parent with a blood cancer, and whether the caregiver stories in the program were authentic and relatable. These items were measured on a 5-point Likert scale, with 5 as the highest score (ie, meets all needs, highly authentic, highly relatable*).* As a further measure of acceptability, we assessed usability by asking about the type of device and type of browser they used. Participants were also asked if they encountered any problems when navigating the web-based program, and if so, they were asked to briefly describe them in an open-ended question.

### Statistical Analysis

We used SPSS (version 28; IBM Corp) to calculate descriptive statistics (frequencies, means, and SDs) for the demographics, the feasibility data, and the acceptability data consisting of the AIM scale, usability questions, and course evaluation items.

### Results

A total of 34 caregivers consented to the study and completed the preprogram survey. Of these, 30 (88%) completed the intervention and the postprogram survey. All 30 participants completed the study within the 2-week time period and 28/30 (93%) completed the postprogram survey within the 1-week time period, meeting our a priori standard of feasibility.

Demographics of the 30 participants are shown in Table 1. The average age of caregivers was 45.6 (SD 11.4; range 24-67) years, and the average age of their parents was 73.5 (SD 9.1; range 57-89) years. Most caregivers (22/30, 73%) were the daughter, stepdaughter, or daughter-in-law of the person for whom they provided care. Participants were asked to select all the races that applied to them. A total of 80% (n=24) of participants reported their race as White, 13% (n=4) as Black or African American, 17% (n=5) as Asian, and 3% (n=1) as Native Hawaiian or Pacific Islander. The majority (25/30, 83%) reported their ethnicity as non-Hispanic, with 17% (n=5) reporting as Hispanic. About half (57%) of participants were employed full time, while the remaining caregivers were either employed part time, retired, self-employed, or not employed. Myeloma (n=11, 37%) and leukemia (n=9, 30%) were the most common types of blood cancer reported.

Prior to the study, and as noted above, we set our acceptability threshold as an average score of 4 on the AIM items. As shown in Table 2, participants found the *Healthy Communication Practice* intervention to be acceptable using the AIM scale (mean 4.48, SD 0.67). Most participants indicated that it was clear how to progress through the program (mean 4.71, SD 0.53) and did not report encountering any problems using the web-based program (n=26, 84%). Most (n=18, 60%) solely used a computer (laptop or desktop) to complete the program, followed by a smartphone (n=4, 13%). The remainder used only a tablet or a combination of devices (eg, smartphone and computer).

**Table 2:** Acceptability of intervention measure.

Items	Rating, mean (SD)
The *Healthy Communication Practice* program meets my approval	4.53 (0.63)
The *Healthy Communication Practice* program is appealing to me	4.40 (0.86)
I like the *Healthy Communication Practice* program	4.45 (0.68)
I welcome the *Healthy Communication Practice* program	4.58 (0.62)
Overall rating	4.48 (0.67)

As shown in Table 3, participants felt the content met the communication needs of caregivers (mean 4.58, SD 0.62) and found that the program met their needs as a caregiver of a parent with a blood cancer (mean 4.40, SD 0.72). In their open-ended feedback responses, they described the program as “an eye-opener,” “very helpful,” and “an excellent learning experience.” A participant noted, “appreciate you taking the time to do this. No one really understands what caregivers go through until they are thrust into the position. It is challenging and can break you in ways you never expected. So, thank you for shining a light and helping with coping mechanisms.”

**Table 3:** Program evaluation.

Items	Rating, mean (SD)
How well does the content of the program meet the communication needs of a caregiver of a parent with a blood cancer?	4.58 (0.62)
How well do you feel the course met your needs as a caregiver of a parent with a blood cancer?	4.39 (0.72)
Please rate the authenticity of the caregiver stories	4.19 (0.75)
Please rate how well you could relate to the caregiver stories	4.32 (0.98)

Participants also specified how the program met their needs by teaching them skills for communicating with clinicians including navigating triadic communication (eg, “to ask permission from my mom before I jump in and start asking questions or speak for her or about her with her physicians. I never realized how important that can be”). They also described learning family communication skills in being open and supportive (eg, “showing up and listening, validating... how to lead the family, as lead caretaker... It’s super helpful showing me how to show up better, for everyone, and even myself”). Furthermore, participants reported in their open-ended feedback that it was “easy to identify with” the caregiver stories. They also evaluated the caregiver stories featured throughout the program as authentic (mean 4.19, SD 0.75) and indicated they could relate to the caregiver stories (mean 4.32, SD 0.98).

In addition, participants reported one area for improvement. Although they found the stories relatable, some indicated that it would be helpful to see narratives of more challenging family dynamics (eg, challenges with in-laws, “dysfunctional” relationships, noncooperative parents). They also mentioned it would be helpful to complete the program earlier in their caregiving experience.

## Discussion

### Principal Findings

We developed a web-based, interactive video-based communication training intervention, *Healthy Communication Practice,* for adult children caring for a parent with a blood cancer. This intervention was developed and tailored to this distinct caregiver type and disease context to ensure their unique communication skills needs were met (G Fennell, PhD, unpublished data, 2007). Piloting the intervention among adult child caregivers of a parent currently or recently in treatment (completed within the last year), we found the intervention to be feasible, as the majority of consented caregivers completed the study (ie, all participants completed the intervention within 2 weeks, and the majority completed the posttraining survey within 1 week). Furthermore, the intervention was evaluated as acceptable by the participants. They reported the program was easy to navigate and met their needs, and that the narratives were authentic and relatable.

The training was engineered to work on a computer, tablet, or smartphone. Although the training was designed to work across technological platforms, most participants reported using a computer to complete the program. Only 4 participants used a smartphone to complete the entire program. The nature of the communication skills training within the program may have felt more comfortable for participants to complete it on a computer given the videos, audio narratives, and interactive activities. Future research should investigate why participants may have chosen to use a computer, and how the program may be better adapted as an app for mobile phone use. Overall, the choice device did not seem to deter participants from completing the program as almost all participants completed it.

### Comparison With Prior Work

When midlife caregivers juggle more roles like caring for a diagnosed parent and caring for children, they likely experience more burden and have a heightened need for supportive interventions like the *Healthy Communication Practice*. In our study, the number of participants reporting full-time employment (n=17, 57%) reflects national estimates for all caregivers (61%) [33]. The majority of caregivers in our study (n=18, 60%) reported having no children. Of those who did have children, only 6 caregivers (20%) had children under the age of 18 years, even though the majority of caregivers were in the earlier phase of midlife (ie, their average age was 46 years), a phase of adulthood in which we would expect caregivers to be responsible for caring for both younger and older generations. However, adult children who have competing roles and responsibilities like parenthood are less likely to become their parent’s caregiver [34,35], particularly when extensive care is needed [36] (ie, care is delegated to childless adult children). An estimated 26% of family cancer caregivers nationally have a child or grandchild under the age of 18 years living with them [37]. Given our sample characteristics, it may have been easier for caregivers who are juggling fewer roles or coping with less caregiving burden to complete this communication skills intervention. Further research should explore ways to reach those with a heightened caregiving load and the best technologies for delivery to further enhance caregivers’ ease of completion.

The *Healthy Communication Practice* program is innovative primarily because of its focus on communication in the context of cancer caregiving. A recent systematic review on cancer caregiving interventions [28] found 33 papers on cancer caregiving interventions, none with a primary focus on communication. As caregiving is enacted primarily through communication, it is critical to address this with cancer caregivers. Other interventions focus primarily on concepts and tasks such as mindfulness [38,39]; stress management [40]; patient symptom management [40]; and topics such as goal setting, planning, accessing family support services, and building problem-solving skills [41]. Our previous research has shown that adult child caregivers’ families who communicate more openly report less caregiver burden, better clinical interaction skills, and better perceived quality of the clinical interaction [17]. Interventions that help family caregivers hone their communication skills are a critical component to supporting caregivers as they navigate the difficulties of caring for a loved one with a blood cancer.

### Limitations

Limitations of our study include a small sample size. In addition, our study design lacked a control group. A further weakness of our sample is selection bias. Due to our recruitment through large national patient advocacy organizations, we likely recruited those who were already motivated to pursue caregiver resources. In addition, as mentioned above, we recruited more participants without children than with children, which may not fully represent midlife adult child caregivers.

### Conclusions

The innovative *Healthy Communication Practice* program is feasible and acceptable in a population of caregivers of a parent with a blood cancer. Future opportunities exist to establish the efficacy of the program and adapt it to other disease caregiving contexts (eg, dementia) and familial contexts (eg, spouses).

In order to establish the efficacy of the program, future research should include a randomized controlled trial of the intervention, testing its impact on both caregiver and patient short- and long-term health outcomes (eg, psychological, relational, and physical well-being; caregiving burden) as well as communication outcomes (eg, increased willingness to communicate with clinicians and family members, more open communication in the family, clinical communication skills engagement). Additionally, future research on this type of intervention should also explore downstream effects of improving caregivers’ communication skills on patient outcomes.

The *Healthy Communication Practice* program could be adapted to more caregiver groups, and thus tailored to recognize and address their distinct needs and experiences in different caregiver relationships (eg, spouses), various age groups (eg, young adults), and other cancer types (eg, breast cancer). If preliminary research in these contexts demonstrates similar core areas in terms of communication skills development needs (eg, navigating web-based and clinical communication; open and supportive family communication), the core concepts of *Healthy Communication Practice* could remain and be tailored with new narratives and video scenarios that reflect the dynamics of the targeted relationships, age group, and disease context. This might also include less functional or more tense relational dynamics to promote narrative transportation. Future research could also explore the efficacy of including a booster 3-6 months following the training to provide caregivers with a reminder of the skill set they learned and offer continued encouragement to enact these communication strategies in their day-to-day lives.

## Appendix

Multimedia Appendix 1 Healthy Communication Practice program screenshots.

## Abbreviations

AIM: Acceptability of Intervention Measure
NIH: National Institutes of Health

## References

[1] Bachner YG, Karus DG, Raveis VH (2009). Examining the social context in the caregiving experience: correlates of global self-esteem among adult daughter caregivers to an older parent with cancer. J Aging Health.

[2] Kent EE, Rowland JH, Northouse L, Litzelman K, Chou WS, Shelburne N, Timura C, O'Mara A, Huss K (2016). Caring for caregivers and patients: research and clinical priorities for informal cancer caregiving. Cancer.

[3] Treanor CJ (2020). Psychosocial support interventions for cancer caregivers: reducing caregiver burden. Curr Opin Support Palliat Care.

[4] Litzelman K (2019). The unique experience of caregivers based on their life stagerelationship to the patient. Cancer Caregivers.

[5] Bluethmann SM, Mariotto AB, Rowland JH (2016). Anticipating the “silver tsunami”: prevalence trajectories and comorbidity burden among older cancer survivors in the United States. Cancer Epidemiol Biomarkers Prev.

[6] Bernard LL, Guarnaccia CA (2003). Two models of caregiver strain and bereavement adjustment: a comparison of husband and daughter caregivers of breast cancer hospice patients. Gerontologist.

[7] Kim Y, Baker F, Spillers RL (2007). Cancer caregivers' quality of life: effects of gender, relationship, and appraisal. J Pain Symptom Manage.

[8] Spillers RL, Wellisch DK, Kim Y, Matthews A, Baker F (2008). Family caregivers and guilt in the context of cancer care. Psychosomatics.

[9] Fenton A, Keating NL, Ornstein KA, Kent EE, Litzelman K, Rowland JH, Wright AA (2021). Comparing adult child and spousal caregiver burden and potential causes. JCO.

[10] Dionne-Odom JN, Currie ER, Johnston EE, Rosenberg AR (2019). Supporting family caregivers of adult and pediatric persons with leukemia. Semin Oncol Nurs.

[11] Pailler ME, Johnson TM, Kuszczak S, Attwood KM, Zevon MA, Griffiths E, Thompson J, Wang ES, Wetzler M (2016). Adjustment to acute leukemia: the impact of social support and marital satisfaction on distress and quality of life among newly diagnosed patients and their caregivers. J Clin Psychol Med Settings.

[12] Howlader N, Noone A, Krapcho M, MIller D, Brest A, Yu M (2019). SEER Cancer Statistics Review, 1975-2017.

[13] (2019). Cancer facts and figures. American Cancer Society.

[14] Fisher CL, Mullis MD, Kastrinos A, Wollney E, Weiss ES, Sae-Hau M, Bylund CL (2021). "Home wasn't really home anymore": Understanding caregivers' perspectives of the impact of blood cancer caregiving on the family system. Support Care Cancer.

[15] Fisher C, Wright K, Hampton C, Vasquez T, Kastrinos A, Applebaum A, Sae-Hau M, Weiss ES, Lincoln G, Bylund CL (2021). Blood cancer caregiving during COVID-19: understanding caregivers' needs. Transl Behav Med.

[16] Kastrinos AL, Fisher CL, Mullis MD, Wollney E, Sae-Hau M, Weiss ES, Bylund CL (2021). A lifespan approach to understanding family caregiver experiences of a blood cancer diagnosis. Pall Supp Care.

[17] Campbell-Salome G, Fisher CL, Wright KB, Lincoln G, Applebaum AJ, Sae-Hau M, Weiss ES, Bylund CL (2022). Impact of the family communication environment on burden and clinical communication in blood cancer caregiving. Psychooncology.

[18] Paige SR, Stellefson M, Krieger JL, Anderson-Lewis C, Cheong J, Stopka C (2018). Proposing a transactional model of ehealth literacy: concept analysis. J Med Internet Res.

[19] Paige SR, Stellefson M, Krieger JL, Miller MD, Cheong J, Anderson-Lewis C (2019). Transactional ehealth literacy: developing and testing a multi-dimensional instrument. J Health Commun.

[20] Bylund CL, Gueguen JA, D'Agostino TA, Imes RS, Sonet E (2009). Cancer patients' decisions about discussing internet information with their doctors. Psychooncology.

[21] Koerner AF, Fitzpatrick MA (2002). Toward a theory of family communication. Commun Theory.

[22] Hinyard LJ, Kreuter MW (2007). Using narrative communication as a tool for health behavior change: a conceptual, theoretical, and empirical overview. Health Educ Behav.

[23] Rolland JS (2005). Cancer and the family: an integrative model. Cancer.

[24] Fisher CL, Nussbaum JF (2015). Maximizing wellness in successful aging and cancer coping: the importance of family communication from a socioemotional selectivity theoretical perspective. J Fam Commun.

[25] Mayer RE (2014). Cognitive Theory of Multimedia Learning. The Cambridge Handbook of Multimedia Learning.

[26] Cohen S, Wills TA (1985). Stress, social support, and the buffering hypothesis. Psychol Bull.

[27] Blok AC, Blonquist TM, Nayak MM, Somayaji D, Crouter SE, Hayman LL, Colson YL, Bueno R, Emmons KM, Cooley ME (2018). Feasibility and acceptability of "healthy directions" a lifestyle intervention for adults with lung cancer. Psychooncology.

[28] Ugalde A, Gaskin CJ, Rankin NM, Schofield P, Boltong A, Aranda S, Chambers S, Krishnasamy M, Livingston PM (2019). A systematic review of cancer caregiver interventions: appraising the potential for implementation of evidence into practice. Psychooncology.

[29] Abbott JH (2014). The distinction between randomized clinical trials (RCTs) and preliminary feasibility and pilot studies: what they are and are not. J Orthop Sports Phys Ther.

[30] Heynsbergh N, Heckel L, Botti M, Livingston PM (2018). Feasibility, useability and acceptability of technology-based interventions for informal cancer carers: a systematic review. BMC Cancer.

[31] Proctor E, Silmere H, Raghavan R, Hovmand P, Aarons G, Bunger A, Griffey R, Hensley M (2011). Outcomes for implementation research: conceptual distinctions, measurement challenges, and research agenda. Adm Policy Ment Health.

[32] Weiner BJ, Lewis CC, Stanick C, Powell BJ, Dorsey CN, Clary AS, Boynton MH, Halko H (2017). Psychometric assessment of three newly developed implementation outcome measures. Implement Sci.

[33] (2020). Caregiving in the United States 2020. AARP and National Alliance for Caregiving.

[34] Pillemer K, Suitor JJ (2014). Who provides care? A prospective study of caregiving among adult siblings. Gerontologist.

[35] Cacciamani GE, Aron M, Gill I, Desai M (2020). Re: Oncological outcome according to attainment of pentafecta after robot-assisted radical cystectomy in patients with bladder cancer in the multicentre KORARC database. BJU Int 2020 July 18. DOI: 10.1111/ bju.15178. BJU Int.

[36] Henretta J, Soldo B, Van Voorhis MF (2011). Why do families differ? Children's care for an unmarried mother. J Marriage Fam.

[37] Cancer caregiving in the U.S.: an intense, episodic, and challenging care experience. NAFC.

[38] (2020). Partnership. National Cancer Institute.

[39] Dragomanovich HM, Dhruva A, Ekman E, Schoenbeck KL, Kubo A, Van Blarigan EL, Borno HT, Esquivel M, Chee B, Campanella M, Philip EJ, Rettger JP, Rosenthal B, Van Loon K, Venook AP, Boscardin C, Moran P, Hecht FM, Atreya CE (2021). Being Present 2.0: online mindfulness-based program for metastatic gastrointestinal cancer patients and caregivers. Glob Adv Health Med.

[40] Al Daken LI, Ahmad MM (2018). The implementation of mindfulness-based interventions and educational interventions to support family caregivers of patients with cancer: A systematic review. Perspect Psychiatr Care.

[41] Hendrix CC, Bailey DE, Steinhauser KE, Olsen MK, Stechuchak KM, Lowman SG, Schwartz AJ, Riedel RF, Keefe FJ, Porter LS, Tulsky JA (2016). Effects of enhanced caregiver training program on cancer caregiver's self-efficacy, preparedness, and psychological well-being. Support Care Cancer.

