# Supplementary material for: Improving Clinical and Family Communication for Adult Child Caregivers of a Parent With a Blood Cancer: Single-Arm Pre-Post Pilot Intervention
Source: JMIR Cancer. 2022 Jul 5;8(3):e38722. doi: 10.2196/38722 (PMC9297135; doi:10.2196/38722)
Supplement: Multimedia Appendix 1 [file cancer_v8i3e38722_app1.pdf]

Healthy Communication Practice™

# Caring for a Parent with a Blood Cancer

Welcome to the Healthy Communication Practice™ Program for Caregivers of a Parent with a Blood Cancer. This two-part program is designed to support the important work you are doing as you care for your parent with a blood cancer. We will present key concepts and skills that can be useful as you navigate online cancer information, communicate with doctors, and facilitate communication within your family. Throughout the two-part program, we will introduce you to caregivers who share stories and demonstrate the skills and concepts we are presenting. We estimate it will take approximately 45 minutes to go through each part of the program. Thank you for joining us!

MEET THE TEAM

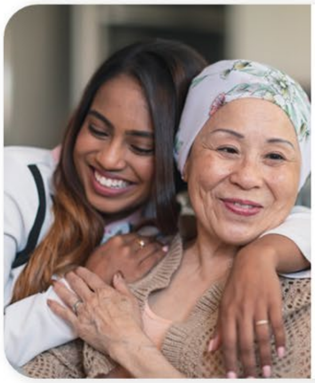

## Part I: Navigating Cancer Information in Online and Clinical Settings

about 45 Minutes    0% Complete

We will describe skills for evaluating online health information, communicating online with others about health information, and communicating with doctors about online health information. Then, we will look at key skills you can use to support your parent in their clinical visits.

GO TO PART I

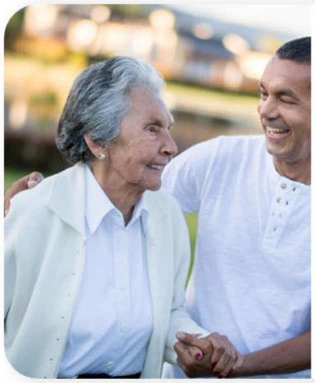

## Part II: Facilitating Open and Supportive Communication In the Family

about 45 Minutes    0% Complete

We will explore the importance of finding meaning in your caregiving role as well as how to strengthen your family relationships with open and supportive communication skills. Then, we will look at how to use these skills when navigating relational challenges and difficult but important caregiving conversations.

GO TO PART II

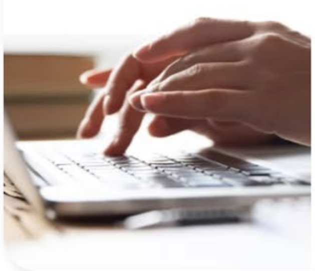

## Caregiver Support Resources

Thank you for completing the Healthy Communication Practice Program™. Here is a PDF that you can download or print that contains a summary of the skills taught in the program. Please see below for additional caregiver support resources from The Leukemia & Lymphoma Society® (LLS).

1. LLS offers a Caregiver Workbook that is a guide to use throughout your time as a blood cancer patient's caregiver. Caregivers may order a copy of the workbook by calling The LLS Information Resource Center (800) 955-4572.
2. For other caregiver resources and information, please see: https://www.lls.org/Caregiver

# Meet the Team

We are an interdisciplinary team of researchers whose work focuses on understanding and improving communication in cancer care for patients, caregivers, and their family. In this program, we will share with you what we have learned in our research that can be helpful to you in the important role you play as a caregiver for a parent with cancer.

RETURN HOME

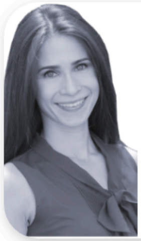

## Allison J. Applebaum

Allison J. Applebaum, PhD, is Associate Attending Psychologist and Director of the Caregivers Clinic in the Department of Psychiatry and Behavioral Sciences at Memorial Sloan Kettering Cancer Center (MSK). Dr. Applebaum's research and clinical practice is focused on promoting the psychological and physical well-being of caregivers of patients with cancer.

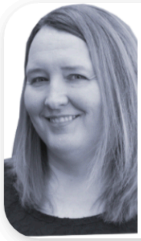

## Carma L. Bylund

Carma L. Bylund, PhD, is Professor in the College of Journalism and Communications and College of Medicine at the University of Florida. Dr. Bylund's grant funded research program focuses on improving communication in cancer care. She is the author of more than 100 peer-reviewed publications and two edited books on healthcare communication.

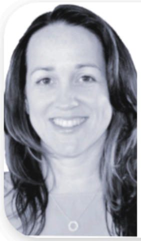

## Carla L. Fisher

Carla L. Fisher, PhD, is Associate Professor in the College of Journalism and Communications and Center for Arts in Medicine at the University of Florida. Dr. Fisher's grant-funded research prioritizes helping families coping with cancer. She has published 70 peer-reviewed publications, including two books on family communication and health.

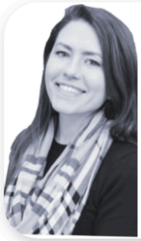

## Samantha Paige

Samantha Paige, PhD, MPH, CHES® is a Research Associate in the College of Journalism and Communications at the University of Florida. Dr. Paige studies how people use technology for health. She has published nearly 50 peer-reviewed publications on topics related to patient education, eHealth literacy, and new communication technologies.

Healthy Communication Practice™

**Part I Overview**

- Introduction
- Critical eHealth Literacy
  - Marco's Story Chapter 1
  - HOPE Activity
- Communicative eHealth Literacy
  - Marco's Story Chapter 2
  - Responding to Marco's Cousin
- Clinical eHealth Literacy
  - Marco's Story Chapter 3
- Communication with Doctors
  - Present Information
  - Ask Questions
  - Check Understanding
  - Express Concerns
  - State Preferences

**Current Progress**

**Introduction**

# Navigating Cancer Information in Online and Clinical Settings

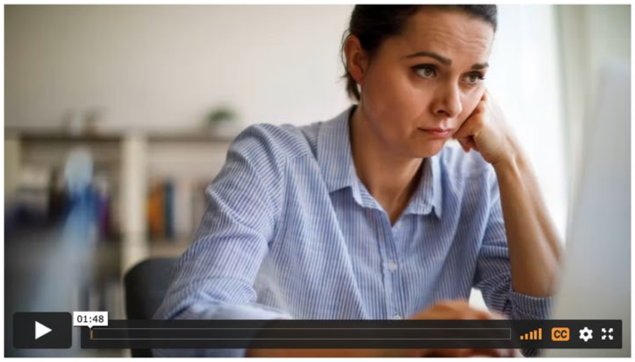


Next, we will introduce the HOPE model, a tool to help evaluate the credibility of online health information.

The **NEXT** and **PREVIOUS** page buttons can help you move through the program.

Healthy Communication Practice™

**Part I Overview**

- Introduction
- Critical eHealth Literacy
  - Marco's Story Chapter 1
  - HOPE Activity
- Communicative eHealth Literacy
  - Marco's Story Chapter 2
  - Responding to Marco's Cousin
- Clinical eHealth Literacy
  - Marco's Story Chapter 3
- Communication with Doctors
  - Present Information
  - Ask Questions
  - Check Understanding
  - Express Concerns
  - State Preferences

**Current Progress**

**Critical eHealth Literacy**

# Critically Evaluating Online Health Information: The HOPE Model

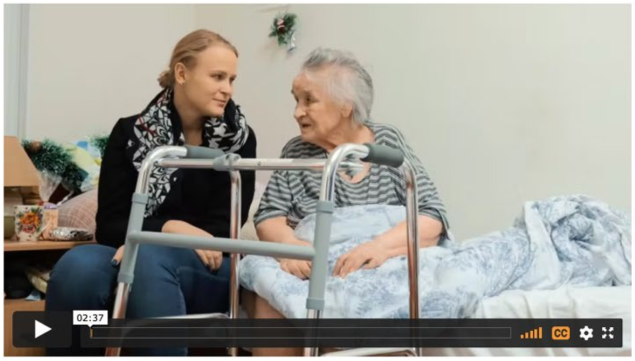


Next, we will further explore the HOPE model by meeting Marco who cares for his father who has leukemia.

**Part I Overview**

- Introduction
- Critical eHealth Literacy
  - Marco's Story Chapter 1
  - HOPE Activity
- Communicative eHealth Literacy
  - Marco's Story Chapter 2
  - Responding to Marco's Cousin
- Clinical eHealth Literacy
  - Marco's Story Chapter 3
- Communication with Doctors
  - Present Information
  - Ask Questions
  - Check Understanding
  - Express Concerns
  - State Preferences

**Current Progress**

**Communicative eHealth Literacy**

# Communicating with Others about Online Health Information

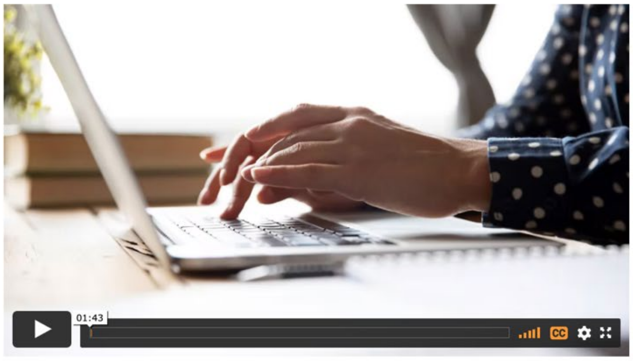


Next, let's return to Marco and see how he communicates with his cousin who shares some online health information.

Healthy Communication Practice™

**Part I Overview**

- Introduction
- Critical eHealth Literacy
  - Marco's Story Chapter 1
  - HOPE Activity
- Communicative eHealth Literacy
  - Marco's Story Chapter 2
  - Responding to Marco's Cousin
- Clinical eHealth Literacy
  - Marco's Story Chapter 3
- Communication with Doctors
  - Present Information
  - Ask Questions
  - Check Understanding
  - Express Concerns
  - State Preferences

**Current Progress**

**Clinical eHealth Literacy**

# Communicating with Doctors about Online Health Information

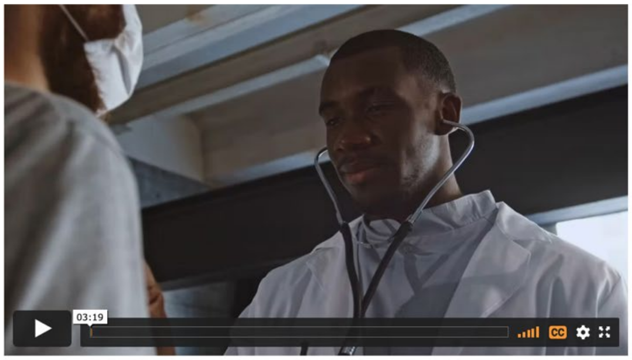


Next, we continue with Marco's journey to access the best health information for his father by examining how he might bring up online health information to doctors who take different approaches to online information.

Healthy Communication Practice™

**Part I Overview**


- Introduction
- Critical eHealth Literacy
  - Marco's Story Chapter 1
  - HOPE Activity
- Communicative eHealth Literacy
  - Marco's Story Chapter 2
  - Responding to Marco's Cousin
- Clinical eHealth Literacy
  - Marco's Story Chapter 3
- Communication with Doctors
  - Present Information
  - Ask Questions
  - Check Understanding
  - Express Concerns
  - State Preferences


**Current Progress**

**Communication with Doctors**

## Using the PACES Approach to Support Your Parent's Communication with Doctors

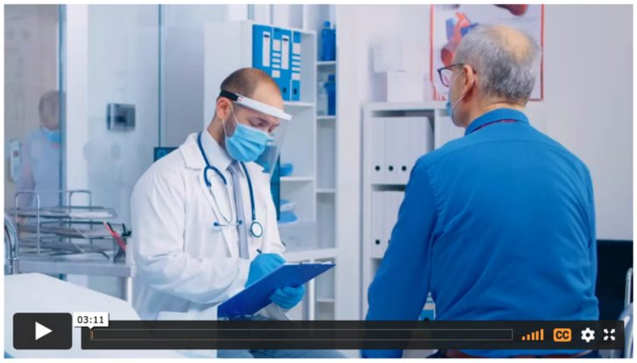


Next, let's see how Anne helps her mother present information to the doctor.

Healthy Communication Practice™

**Part II Overview**


- Introduction
- Connecting to Meaning
  - Caregivers' Stories
  - Reflection Activity 1
- Strengthening Family Bonds
  - Caregivers' Stories
  - Reflection Activity 2
- Challenges and Communication Skills
  - Caregivers' Stories
  - Strategies Activity
- Practicing Skills
  - Sharing Caregiving Responsibilities
  - Future Uncertainty and Mortality
  - Distressing Emotions and Concerns
- Conclusion
- Resources
- Acknowledgments


**Current Progress**

**Connecting to Meaning**

## Finding Meaning in Your Caregiving Role

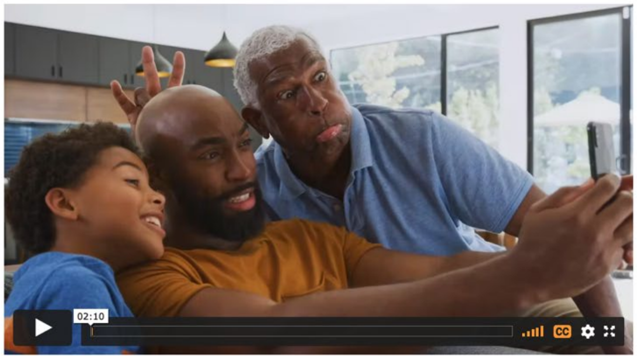


Next, we will hear how caregivers have found meaning in their role.

Part II Overview

Introduction
Connecting to Meaning
- Caregivers' Stories
- Reflection Activity 1
Strengthening Family Bonds
- Caregivers' Stories
- Reflection Activity 2
Challenges and Communication Skills
- Caregivers' Stories
- Strategies Activity
Practicing Skills
- Sharing Caregiving Responsibilities
- Future Uncertainty and Mortality
- Distressing Emotions and Concerns
Conclusion
Resources
Acknowledgments

Current Progress

Strengthening Family Bonds

## The Ties That Bind: Creating a Healthy Communication Practice

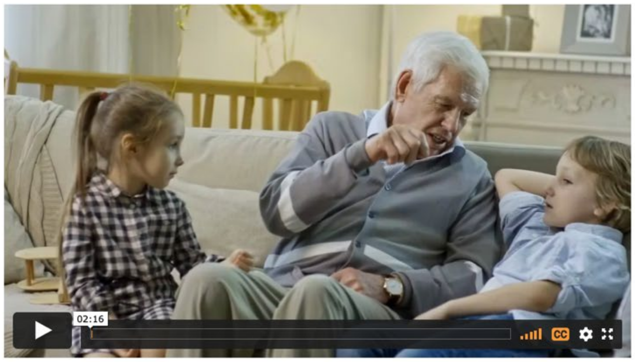


Next, we will hear about caregivers practicing open and supportive communication.

Healthy Communication Practice™

Part II Overview

Introduction
Connecting to Meaning
- Caregivers' Stories
- Reflection Activity 1
Strengthening Family Bonds
- Caregivers' Stories
- Reflection Activity 2
Challenges and Communication Skills
- Caregivers' Stories
- Strategies Activity
Practicing Skills
- Sharing Caregiving Responsibilities
- Future Uncertainty and Mortality
- Distressing Emotions and Concerns
Conclusion
Resources
Acknowledgments

Current Progress

Challenges and Communication Skills

## Identifying Challenges: Relational Tensions and Difficult Topics

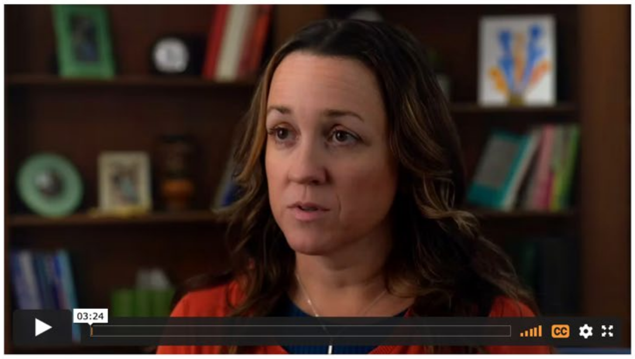


Next, we will hear how caregivers have navigated challenging conversations.

**Part II Overview**

- Introduction
- Connecting to Meaning
  - Caregivers' Stories
  - Reflection Activity 1
- Strengthening Family Bonds
  - Caregivers' Stories
  - Reflection Activity 2
- Challenges and Communication Skills
  - Caregivers' Stories
  - Strategies Activity
- Practicing Skills
  - Sharing Caregiving Responsibilities
  - Future Uncertainty and Mortality
  - Distressing Emotions and Concerns
- Conclusion
- Resources
- Acknowledgments

**Current Progress**

**Practicing Skills**

## Practicing Open and Supportive Communication Skills: Remembering "TIES" That Bind

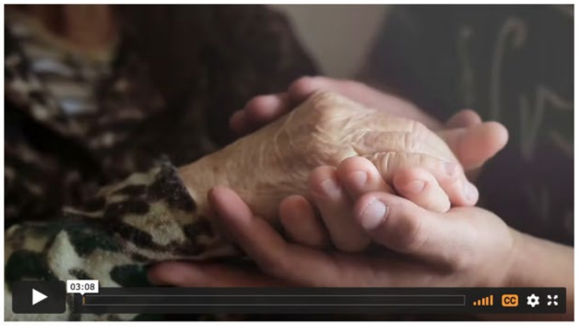


Next, watch at least one of the three videos to observe how to enact open and supportive communication skills to navigate one of the challenging topics.

- Talking to Siblings About Sharing Caregiving Responsibilities
- Talking to a Parent About Future Uncertainty and Mortality
- Talking to a Parent About Distressing Emotions and Concerns

Healthy Communication Practice™

**Part II Overview**

- Introduction
- Connecting to Meaning
  - Caregivers' Stories
  - Reflection Activity 1
- Strengthening Family Bonds
  - Caregivers' Stories
  - Reflection Activity 2
- Challenges and Communication Skills
  - Caregivers' Stories
  - Strategies Activity
- Practicing Skills
  - Sharing Caregiving Responsibilities
  - Future Uncertainty and Mortality
  - Distressing Emotions and Concerns
- Conclusion
- Resources
- Acknowledgments

**Current Progress**

**Conclusion**

## Conclusion

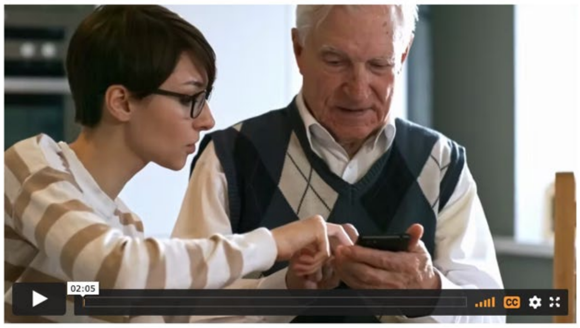


Thank you for participating in the Healthy Communication Practice™ Program. For additional resources, including a summary of the skills taught in the program, please click "Next Page" for the "Caregiver Support Resources" section of the program.

**Part II Overview**

- Introduction
- Connecting to Meaning
  - Caregivers' Stories
  - Reflection Activity 1
- Strengthening Family Bonds
  - Caregivers' Stories
  - Reflection Activity 2
- Challenges and Communication Skills
  - Caregivers' Stories
  - Strategies Activity
- Practicing Skills
  - Sharing Caregiving Responsibilities
  - Future Uncertainty and Mortality
  - Distressing Emotions and Concerns
- Conclusion
- Resources
- Acknowledgments

**Current Progress**

**Resources**

# Caregiver Support Resources

Thank you for completing the Healthy Communication Practice Program™. Here is a PDF that you can download or print that contains a summary of the skills taught in the program. Please see below for additional caregiver support resources from The Leukemia & Lymphoma Society® (LLS).

1. LLS offers a FREE Caregiver Workbook that is a guide to use throughout your time as a blood cancer patient's caregiver. Caregivers may order a copy of the workbook by calling The LLS Information Resource Center (800) 955-4572.
2. For other caregiver resources and information, please see: https://www.lls.org/Caregiver

Healthy Communication Practice™

**Part II Overview**

- Introduction
- Connecting to Meaning
  - Caregivers' Stories
  - Reflection Activity 1
- Strengthening Family Bonds
  - Caregivers' Stories
  - Reflection Activity 2
- Challenges and Communication Skills
  - Caregivers' Stories
  - Strategies Activity
- Practicing Skills
  - Sharing Caregiving Responsibilities
  - Future Uncertainty and Mortality
  - Distressing Emotions and Concerns
- Conclusion
- Resources
- Acknowledgments

**Current Progress**

**Acknowledgments**

# Special thanks to ...

### Advisory Board

- Kennan DeGruccio
- Skyler Johnson, MD
- Cheryl Martin
- Matthew Matasar, MD
- Martina Murphy, MD
- Maria Sae-Hau, PhD
- Lynn Steele, LSW, OSW-C
- Elisa Weiss, PhD
- Kevin Wright, PhD

### E-Learning Experience Production

- Jason Dean Arnold, EdD
- Domenic Durante, MAE
- Mark Dinsmore
- Jessica Kate Holmer
- Cristian Omar Jimenez
- Steven Zill
- Kayla Sharp

### Additional Filming

- Donald Bruce
- Michael DeGruccio
- Zac Miller
- Sam Palmucci
- Edwin Swisher

### Funding Organizations

- The Leukemia & Lymphoma Society®
- The Carolan Research Institute

### Narrator

- Gregg Jones

### Actors

- Peter Coriaty
- Kennan DeGruccio
- Eileen DeGruccio
- Sandra DeLuca
- Ryan George
- E. Stanley Richardson
- Brijen Shah, MD

### Voice Actors

- Rae Bael
- Courtney Bess
- Junko Cheng
- Anurag Gupta
- Kelly LaBrecque
- Greg Lincoln
- Adam Mark
- Alexis Williams

Students and Trainees in Dr. Bylund's Communication in Healthcare Lab and Dr. Fisher's Family-Health-Lifespan Communication Lab

We dedicate this caregiver program to the memory of Dr. Geraldine Fennell, whose work articulated the importance of effectively developing and delivering tailored support and information to caregivers, especially to subgroups of caregivers with distinct needs.

# Healthy Communication Practice™

## Caring for a parent with a blood cancer

### The HOPE model of evaluating online health information

**H**onest and trustworthy
**O**bjective
**P**ractical
**E**xpertise

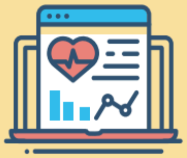

### Communicating with others about online health information

1. Let them know you've read their message
2. Reflect and describe their thoughts
3. Ask their opinion about the quality of information
4. Outline the next steps for confirming whether the information they shared with you is high quality

### Communicating with doctors about online health information

1. State your reason for reading online health information
2. Describe the steps you have taken to ensure you are choosing credible information
3. Acknowledge the doctor's expertise
4. Express appreciation for the doctor's partnership and willingness to listen

## PACES approach for communicating with doctors

**P**resent information

**A**sk questions

**C**heck understanding

**E**xpress concerns

**S**tate preferences

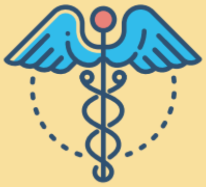

## 4 Skills for supporting your parent in communicating with their doctor

1. Introduce yourself at the beginning of the visit
2. Ask permission from your parent to share information or ask questions
3. Check with your parent to make sure what you say is correct
4. Encourage your parent to use good communication skills

---

## TIES approach to practicing open and supportive family communication

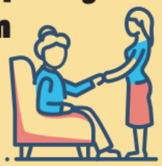

**T**ake the lead

**I**nitiate the conversation

**E**xplain the importance of the conversation

**S**ustain supportive and open communication in the moment and with ongoing conversations

## Skills for communicating openness and support with family

1. Be direct and express your caregiving needs
2. Address the benefits to discussing the issue
3. Listen and validate your loved one's feelings
4. Show love and concern
